# A Modified Triangular Arthroscopic Suture-Based Fixation for Tibial Eminence Fractures: Technique, Outcome and Literature Review

**DOI:** 10.3390/jcm13164950

**Published:** 2024-08-22

**Authors:** Guy Morag, Gil Rachevski, Oleg Dolkart, Ehud Rath, Jeremy Dubin, Ofir Chechik, Michael Drexler, Ran Atzmon

**Affiliations:** 1 Tel Aviv Medical Center, Orthopedic Surgery Department, Tel Aviv 6423906, Israel; 2 Assuta Medical Center, Orthopedic Surgery Department, Ashdod 7747629, Israel

**Keywords:** arthroscopy, triangular suture fixation, tibial eminence fractures, intercondylar eminence fractures, Meyers and McKeever classification

## Abstract

**Objectives:** Tibial eminence fractures account for 2% to 5% of all knee injuries. Low-grade fractures, such as Type I, are typically treated conservatively, whereas high-grade fractures, such as Types III and IV, usually require surgical intervention. This paper describes a modified surgical arthroscopic technique, which employs pull-through triangle suture fixation for Type II and Type III arthroscopic intercondylar eminence avulsion fractures. In addition, we examined the efficacy and complication rate compared to the existing literature. **Methods:** Data were prospectively collected for knee arthroscopy surgeries and retrospectively analyzed with a minimum two-year follow-up. Twenty-three consecutive adults underwent arthroscopic treatment of displaced intercondylar Type II and Type III eminence fractures, as evidenced by clinical examination and imaging studies between May/2008 and May/2021. The patient’s knee evaluation was performed using clinical symptoms and physical examination, along with International Knee Documentation Committee (IKDC) questionnaire and Tegner Activity Score. Compared to the literature, post-hoc power was calculated based on the mean Tegner Activity Score in our analysis. **Results:** fifteen females and eight males (mean age 33.9 years, range 19–56 years) were enrolled. The average postoperative follow-up was 35.4 months (27–53). The post-hoc power was 95% confidence in terms of the Tegner Activity Score. The mean ± standard deviation postoperative Tegner Activity Score was 8.2 ± 1.7 (6.8–10.0). Fifteen patients were classified as IKDC A (normal), six as IKDC B (nearly normal), and two as IKDC C (abnormal). The mean IKDC subjective score was 72.7 ± 23 (23–100). Twenty-four patients achieved normal flexion degrees compared with the unaffected side, while one patient achieved a flexion of only 0–90°. The group’s mean flexion range of motion was 123 ± 16° (90–150°). **Conclusion:** This study presents a modified surgical arthroscopic suture fixation technique for tibial eminence fractures. The procedure is relatively simple and requires no more than basic arthroscopy equipment. The clinical and radiographic results indicate that this technique is safe, efficient, enables early initiation of rehabilitation, and has a lower complication rate in a variety of aspects compared with other fixation techniques used for tibial eminence fractures.

## 1. Introduction

Tibial eminence fractures, also known as intercondylar eminence avulsion fractures, are relatively rare, with an incidence of 3/100,000 cases per year. These types of fractures make up 2% to 5% of all knee injuries and are often associated with other intraarticular pathologies, such as meniscal injury, articular cartilage damage, and tibial plateau fractures [1,2,3,4,5]. Meyers and McKeever devised a system to classify these fractures into three main types [6]. Type I fractures involve minimal elevation of the anterior margin of the fragment, while Type II fractures exhibit anterior lifting of one-third to one-half of the tibial eminence from the epiphyseal bed through a posterior hinge. Type III fractures are further divided into Type IIIa and Type IIIb, with the former being characterized by complete displacement of the fragment and the latter by displacement and rotation [6]. Zaricznyj [7] later revised the classification by introducing Type IV, which involves a comminuted avulsion fracture. Treatment options for tibial eminence fractures vary depending on the type of fracture. Nonoperative immobilization is typically recommended for Type I fractures and some Type II fractures, such as those with minimal anterior lifting and non-comminuted fractures. At the same time, open or arthroscopic reduction and internal fixation are suggested for Type III and Type IV fractures to prevent complications such as nonunion, malunion, instability, or loss of knee extension [8,9,10,11,12,13]. The ultimate therapeutic goal is to restore the length and tension of the anterior cruciate ligament (ACL) and prevent further knee laxity.

Several arthroscopic fixation techniques have been reported, such as crossed percutaneous K-wire fixation [14,15], screws fixation [16,17], staple fixation [18,19], suture fixation [20,21,22,23,24,25], and K-wire folded fixation [26]. The advantages of using arthroscopic fixation include less postoperative pain, shorter hospitalization, better cosmetic results, early rehabilitation, and fewer motion complications [27]. The pull-through triangle suture fixation technique is utilized for solely suture fixation during the procedure of attaching the intercondylar eminence by passing it through the base of the Anterior Cruciate Ligament (ACL) insertion, creating a triangular shape fixation with a broad and stable base. This method has several advantages, including the use of primarily absorbable sutures, which promote biological bone healing processes without leaving foreign bodies that require a second surgery for removal.

However, ongoing debate remains regarding the most effective technique for reducing postoperative complications. Additionally, there is not enough evidence in the literature on the effectiveness of the pull-through triangular suture technique for high-grade tibial intercondylar eminence fractures. We hypothesize that a modified surgical arthroscopic technique for the reduction and fixation of tibial eminence fractures using pull-through triangle suture fixation will enable patients to regain their pre-injury range of motion and activity scores while minimizing postoperative complications.

## 2. Materials and Methods

The local institutional review board approved the study and involved a retrospective analysis of prospectively collected data from a single center. All procedures were performed by a single, fellowship-trained knee arthroscopy specialist Between May 2008 and May 2021. The procedures involved twenty-three adult patients who had intercondylar Type II and Type III tibial eminence fractures, according to the Meyers and McKeever classification system [6,7]. Due to the rarity of this type of fracture, the results were compared to the existing literature without using a control group. Following data analysis, the post hoc power analysis using the OpenEpi.com sample size calculator demonstrated a statistical power of 0.80, incorporating an alpha level of 0.05 (95% confidence) to evaluate the Tegner activity scale.

### 2.1. Study Population

The study included 23 consecutive adult patients who underwent arthroscopic treatment of displaced intercondylar fractures. The inclusion criteria consisted of adults with closed physis and displaced intercondylar fractures classified as Meyers and McKeever Types II or higher without significant additional injuries. The exclusion criteria included tibial plateau fractures, nerve or vessel damage, multi-ligament injuries, knee dislocation, and less than 24 months of postoperative follow-up. Preoperatively, the patients were diagnosed based on clinical examinations and imaging studies, including standard anteroposterior and lateral knee radiographs and computed tomography (CT) scans. Magnetic Resonance Imaging (MRI) was not routinely obtained and was reserved for complicated cases where X-ray and CT imaging were insufficient. Anterior instability was assessed through the anterior drawer test and Lachman test. The anterior drawer test was performed at 90° of knee flexion and graded according to the extent of anterior translation (grade I = translation of <5 mm, grade II = translation of 5 to 10 mm, and grade III = translation of >10 mm) that has become an appropriate baseline [28,29]. According to the Meyers and McKeever classification, there were fourteen Type II fractures and nine Type III fractures [6,7]. Type II fractures were treated surgically if they exhibited an anterior lift of 4 mm or more or if the fracture consisted of more than one piece. The causes of the injuries were as follows: Fifteen road traffic and biking accidents, eight skiing accidents, and two falls. Of the patients, 6 had a combination knee injury, including 3 with a meniscal tear, 2 with grade I-II injuries to the medial collateral ligament, and 1 with a grade II injury to the lateral collateral ligament.

### 2.2. Data Acquisition

Data were prospectively retrieved and retrospectively analyzed from clinical and operative notes, and patients were contacted via phone to complete a self-reported complications questionnaire regarding the recurrence of symptoms, current activity level, and satisfaction level. The Tegner activity score was used to evaluate pre and post-surgery activity levels [27,30], and the Lachman test was used to assess knee mobility at the latest follow-up clinic visit [28,29]. The International Knee Documentation Committee (IKDC) subjective Knee Evaluation form was also used to assess symptoms and signs [31,32]. The IKDC categories were graded A (normal), B (nearly normal), C (abnormal), or D (severely abnormal). The healing of fractures was confirmed through lateral radiographs and by the absence of anterior knee pain at the most recent follow-up.

### 2.3. Operative Technique

**Figure 1:**
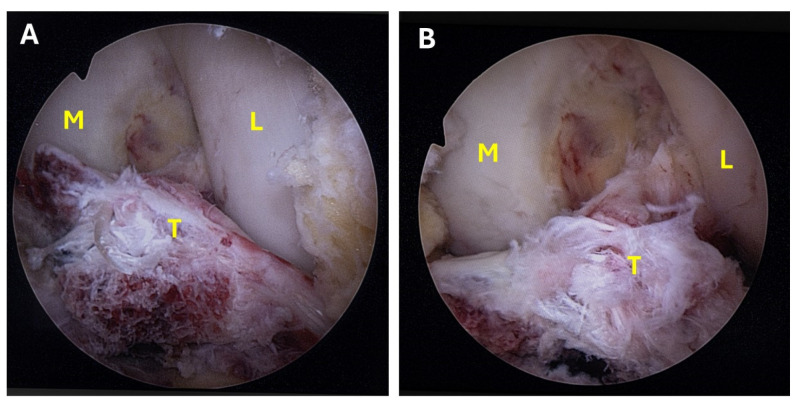
(**A**,**B**) Types III displaced tibial eminence fracture (T = tibial eminence, M = medial condyle, L = lateral condyle).

**Figure 2:**
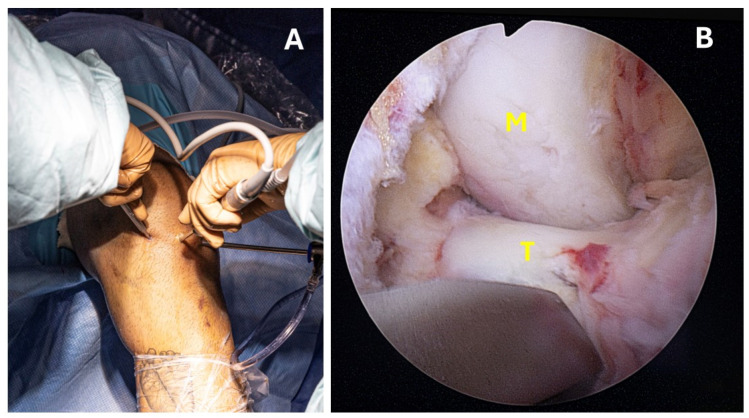
(**A**,**B**) Showing the anatomical reduction of the tibial eminence using a rasp. (**A**) shows the rasp being inserted through the transpatellar portal. (**B**) presents an intra-articular view (T = tibial eminence, M = medial condyle).

The patient was placed supine with a midthigh tourniquet and a leg holder on the ipsilateral side to allow full knee Range Of Motion (ROM). Standard anteromedial and anterolateral portals were created, and a 30° scope and a 4.0 mm probe were used to examine the knee joint and remove debris and other interposed tissues. Concomitant meniscal tears and other lesions were assessed and addressed prior to treating the fracture. In most cases, the transverse intermeniscal ligament was interposed between the superior displaced fragment and the tibia, obstructing accurate reduction. By using a probe from the anteromedial portal and creating an additional transpatellar portal, the ligament was pulled anteriorly and over the tibial eminence fragment (Figure 1A,B), thereby facilitating the achievement and maintenance of reduction (Figure 2A,B).

**Figure 3:**
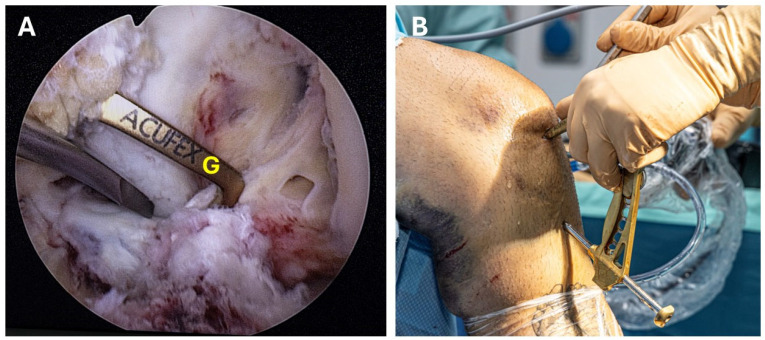
(**A**,**B**) Demonstrating the temporary reduction of the tibial eminence with a rasp and the ACL tibial guide adjusted to 65° located at the center and posterior part of the tibial eminence. (**A**) Intra-articular view. (**B**) Setting of the surgical equipment. (G = ACL tibial guide).

**Figure 4:**
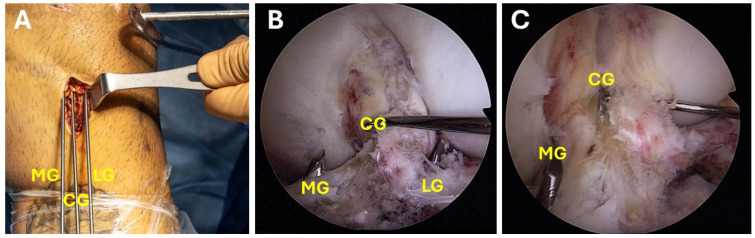
(**A**–**C**) Showing the three 2.4-mm guidewires setup, creating a triangular formation. (**A**) Extra articular guidewires formation, (**B**,**C**) intra articular formation. (MG = medial guidewire, CG = central guidewire, LG = lateral guidewire).

**Figure 5:**
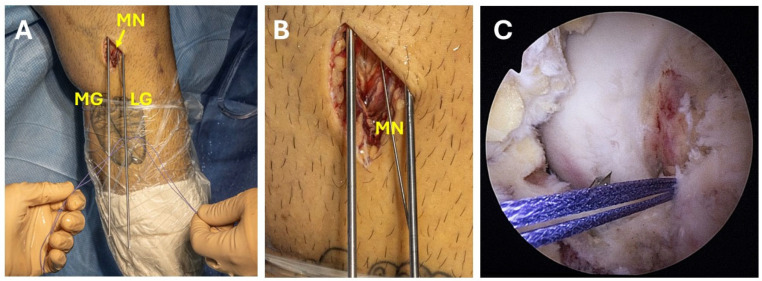
(**A**–**C**) Showing the replacement of the guidewire with the meniscal repair needle; the central guidewire is removed first, followed by the placement of a meniscal needle with two Vicryl 2 sutures attached. (**A**,**B**) central meniscal repair needle loaded with two Vicryl sutures, (**C**) intraarticular view of the two Vicryl sutures (MG = medial guidewire, MN = meniscal needle, LG = lateral guidewires).

**Figure 6:**
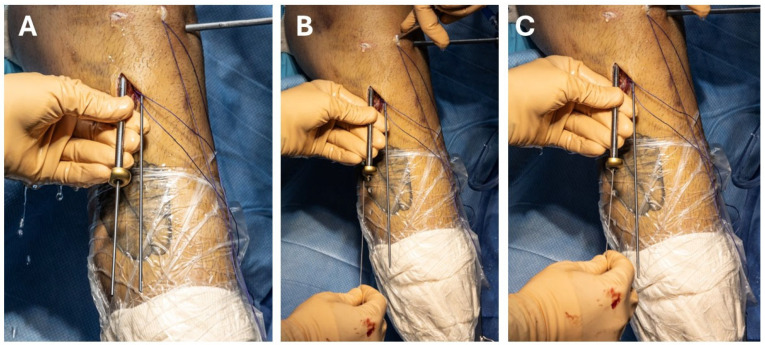
(**A**–**C**): Demonstrating the surgical technique for replacing the guidewire with the meniscal needle. (**A**) First, the ACL hollow bullet is slid over the guidewire, thereby securing the hole. (**B**) Next, the guidewire is removed, and the needle is inserted (**C**).

Initially, the ACL tibial drill guide (Linvatec, Largo, FL, USA) is set at 65° and inserted through the anteromedial portal (Figure 3A,B). Then, a 2 cm anterior oblique incision is made on the anteromedial aspect of the tibia, and a 2.4 mm guidewire is placed centrally through the ACL insertion. Two additional guidewires are then drilled at a 55° angle from the ACL tibial drill guide, one medially and one laterally from the first guide. These guidewires are placed at the anterior aspect of the fragment, specifically at the anteromedial and anterolateral holes, located 1 to 2 mm outside the perimeter of the fracture crater (Figure 4A–C). Next, the guidewires are replaced with meniscal repair needles (Conmed, Utica, NY, USA). These needles are inserted into the joint through the previously drilled holes, as depicted in Figure 5A–C. The central guidewire is removed first, followed by the placement of a meniscal needle with two Vicryl 2 sutures attached. Next, the medial guidewire is removed, and a meniscal needle (or suture passer) equipped with two No. 2 nonabsorbable Ethibond sutures (Ethicon, Somerville, NJ, USA) is introduced through the drill hole. The suture is then pulled out through the transpatellar portal (Figure 6A–C).

**Figure 7:**
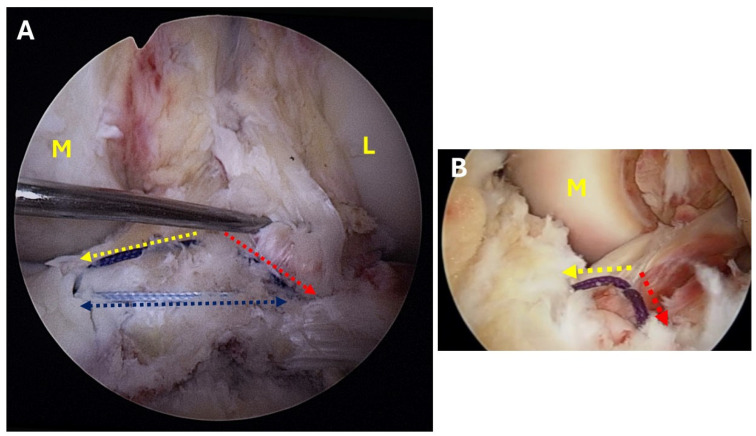
(**A**,**B**) Intraarticular view of the triangular suture configuration, showing the medial and lateral limbs originating from the apex of the triangle (the center of the eminence) and inserted into the medial and lateral drill holes. Additionally, a transverse anterior limb connects these two drill holes. (Yellow arrow = medial limb, Red arrow = lateral limb, Blue arrow = anterior limb, M = medial condyle, L = lateral condyle).

One of the Ethibond sutures serves as a passing suture for one of the Vicryl 2 posterior sutures, passing it through the medial hole. This arrangement causes one suture to extend over the fragment from the middle posterior to the anterior medial portal. Next, the lateral guidewire is removed, and a meniscal needle (or suture passer) carrying a single No. 2 nonabsorbable Ethibond suture is introduced into the joint through the drill hole. The Ethibond suture is then pulled out through the transpatellar portal, where it is used as a passing suture. The Ethibond suture, used as a passing suture, pulls one Vicryl No. 2 posterior suture and another Ethibond No. 2 suture from the medial hole through the lateral portal. This maneuver causes one suture to span over the fragment from the middle-posterior to the anterior-lateral portal while the other crosses from medial to lateral. Consequently, a triangular suture configuration is formed around the fragment. This triangular suture is tightened using a Cobb instrument inserted from the transpatellar portal to reduce the fragment (Figure 7A,B). Finally, after achieving an anatomic reduction of the avulsed fragment, the sutures are tied over the tibial cortex at 30° of knee flexion.

### 2.4. Postoperative Procedure

Post-surgical rehabilitation included a functional locked knee brace in 10° of hyperextension with full weight-bearing as tolerated, using crutches, and administering prophylactic anti-thrombosis treatment with 40 milligrams of Enoxaparin sodium for 6 weeks. Continuous Passive Motion (CPM) therapy was initiated two days postoperatively, starting with 0 to 30° for the first two weeks, followed by a gradual increase of 30° every two weeks. Muscle strengthening exercises included straight leg raising, static quadriceps exercises, and patellar mobilization. After six weeks, patients were allowed to engage in active full range of motion exercises, use a stationary bike, participate in pool exercises, and perform closed chain exercises. Light jogging could commence after 3 to 4 months based on quadriceps strength. Return to sports was permitted after 5 to 6 months, provided that quadriceps strength reached 85% compared to the contralateral side.

## 3. Results

A total of 23 arthroscopic reductions and fixations of displaced Type II and Type III tibial intercondylar eminence fractures were performed in our institution between May 2008 and May 2021. The patient group consisted of fifteen females and eight males, with a mean age of 33.9 years (range 21–56 years). Further demographic data are available in Table 1. The average time gap between injury and surgery was 6.9 days (1–15 days), and the postoperative follow-up for the entire group ranged from 27 to 53 months, with an average of 35.4 months. The postoperative Tegner Lysholm Knee Score had an average of 81.9 (17.4) (range: 68–100). Of the patients, seven were classified as IKDC A, two as IKDC B, and one as IKDC C, with the mean IKDC subjective score for all categories being 72.7 ± 23 (range: 23–100). ROM measurements indicated that 22 patients achieved normal flexion compared to the unaffected side, while one patient achieved only 0–90° of flexion. The group’s mean flexion ROM was 123 ± 16° (range: 90–150°) (Table 1).

Upon final follow-up, the Lachman and drawer tests indicated no evidence of ACL deficiency or impingement. At the six-month follow-up, all patients’ radiological examinations showed complete bone union, with eighteen displaying no signs of elevation. Five patients could not fully recover their range of motion after the operation; among them, four patients underwent manipulation under anesthesia followed by an arthroscopic arthrolysis and scar tissue debridement at three and nine months postoperatively. However, no significant difference was observed between their ROM and that of the remaining patients during the final review. Only one patient opted out of the second surgery and had to contend with a limited range of motion (0–90°). None of the patients experienced hardware disturbance, and no further operation was required to remove the hardware. Additionally, no complications were directly associated with the arthroscopy, and no deep infection or venous thrombosis cases were reported.

## 4. Discussion

The present study introduces a novel arthroscopic surgical technique that employs a triangular suture fixation approach to stabilize a bone fragment while preserving the integrity of the ACL. Previous suturing techniques have utilized a single bone tunnel [19,21], but the innovation of the current technique lies in its triangular configuration and anchoring maneuver, which firmly secures the bony fragment to the tibial spine and evenly distributes tension around it. This approach prevents fragment tilting and movement, allowing immediate full weight-bearing and range of motion. In addition, unlike other Arthroscopic reduction and internal fixation (ARIF) techniques [33], this technique mainly uses absorbable sutures to provide initial fixation and stability until they disintegrate, allowing for natural bone healing without the need for foreign body removal (i.e., metal or screws) in a subsequent surgery due to loosening or impingement.

Several studies have reported a Tegner Lysholm Knee Score similar to ours despite different suture techniques [33,34,35,36]. In their study, Yıldırım et al. [36] utilized an arthroscopic double-loop endobuttton device on 13 patients with type II to Ib tibial eminence fractures. Their postoperative Tegner Lysholm knee score and IKDC scores were 87.1 ± 5.4 and 80.2 ± 4.0, respectively, with negative Lachman and pivot-shift tests at the final follow-up. Chu and colleagues [34] reported an 88.14 Tegner Lysholm Knee Score and IKDC of 84.29 while employing a double-row anchor suture-bridge technique on 7 patients. Finally, Hunter and colleagues [33]. reported on seventeen patients with Meyers and McKeever Type II or III tibial eminence fractures who were treated with arthroscopic suture or screw fixation, and their IKDC scores were evaluated. The authors classified nine patients as IKDC A, seven as IKDC B, and one each as IKDC C and IKDC D, with a mean IKDC subjective score of 70.71 ± 17.56. Similarly, our study observed a comparable distribution of IKDC scores and an average Tegner Lysholm Knee Score outcome of 81.9 and 72.7, respectively. We believe that the somewhat less favorable IKDC outcomes can be attributed to the larger number of patients in our study compared to previous studies, resulting in a more diverse range of results [6,10,26,33,34,35,36,37].

While residual laxity was frequently observed in various studies, it did not have any clinical significance in most cases [9,10,33,35,38]. In a study by Baxter and Wiley [9]. Thirty-two patients with tibial eminence avulsion fractures were divided into surgical and non-surgical groups. The authors observed an average side-to-side difference of 3.5 mm in anterior knee translation laxity, but none of the patients reported knee instability. Similarly, Willis and colleagues [38] used a KT-1000 examination and found that most patients (37 out of 50) did not exhibit symptomatic instability. Gans et al. [10] conducted a meta-analysis and reported positive results for the Lachman and anterior drawer tests in 22.2% of patients with Types I and II fractures and in 60.3% of patients with Types III and IV fractures after fracture treatment. While they were unable to compare suture versus screw fixation due to insufficient data, descriptive statistics suggested the superiority of suture fixation over screw fixation. However, though screw fixation was associated with a higher rate of positive Lachman and anterior drawer tests compared to suture fixation (82.4% vs. 18.8%, respectively), both methods had a similar rate of positive pivot-shift examination, with none of the patients exhibiting pathological laxity above IIB, that would require surgery [10]. Finally, a large systematic review by Osti et al. [35] encompassed 24 studies with various fixation methods, 11 of which were comprised of only suture techniques. The majority of the studies reported good stability with some residual laxity. Likewise, in the current study, we did not observe any patients who reported instability, and there were no indications of post-surgical hardware impingement.

Regaining preoperative range of motion (ROM) is crucial after an ARIF procedure, but several factors can affect the postoperative ROM. These factors include hardware impingement, arthrofibrosis, scar tissue formation, knee effusion, nonunion, malunion, meniscal or ligamentous injury, bony deformity, and others [6,10,26,33,37]. In a study conducted by Faivre et al. [26], eight patients who underwent the ARIF procedure using a Tightrope^®^ device were followed up. Among these patients, three experienced severe difficulties in regaining their preoperative ROM. Two of the three patients underwent manipulation under anesthesia combined with arthroscopic arthrolysis and scar debridement at 2.5 and 6 months after the primary operation. The authors highlighted the importance of initiating early rehabilitation protocol for all eight patients. In their meta-analysis, Gans et al. [10] defined arthrofibrosis as a lack of 10° or more in extension and/or a lack of 25° of flexion or more at three months postoperatively, which persisted despite physical therapy and elimination of other possible causes. Due to the lack of adequate data, a meta-analysis comparing screw versus suture fixation or ARIF versus ORIF was impossible. Therefore, only descriptive statistics were provided. These statistics revealed that arthrofibrosis was observed in 7.1% of patients with Types I and II fractures and in 14.2% of patients with Types III and IV fractures. Of note, no patients in the screw fixation group developed arthrofibrosis, in contrast to 6.3% of those treated with suture fixation. Akin to our study, Osti et al. reported a good to excellent functional range of motion exceeding 120° in their extensive systematic review, which included an analysis of 11 papers on suture fixation techniques [35].

In contrast to the findings of Montgomery et al. [39], where more than 50% of patients who underwent ARIF with nonabsorbable sutures required a second surgery involving manipulation under anesthesia followed by arthroscopic arthrolysis, the current study showed different outcomes. Among the 22 patients in this study, the majority achieved a range of motion comparable to their unaffected contralateral side, with only one patient experiencing limited ROM at the final follow-up. Four patients did require a second surgery for manipulation under anesthesia followed by arthroscopic arthrolysis due to arthrofibrosis. Consistent with the findings of Zhang et al. [40], no significant complications such as vascular or neural injury, infection, or thrombosis were observed during the follow-up period, and radiological assessments confirmed that all fractures had completely healed.

Comparatively, a systematic review by Limone et al. [41] found that arthroscopic suture fixation generally resulted in higher clinical scores but also a higher incidence of arthrofibrosis compared to screw fixation. Arthrofibrosis was more common with suture fixation (*p* < 0.05), whereas screw fixation often required hardware removal due to complications (*p* < 0.001). This aligns with our findings, where patients did not report instability at the final follow-up, and Lachman and drawer tests showed no evidence of ACL deficiency or impingement.

In their review, Hunter et al. [33] presented an arthroscopic fixation technique that involved the utilization of two standard anteromedial and anterolateral knee portals along with a third accessory portal for maintaining reduction. Typically, the accessory portal was either a mid-patellar (transpatellar) or medial portal. In our current technique, we employed a transpatellar portal, which provided a direct and straight trajectory to the triangular knot, thereby decreasing the risk of damaging major vascular structures and reducing postoperative disability.

This study focuses on the adult population with fully developed skeletons, even though this type of fracture is more common in pediatric populations with immature skeletons. However, these fractures occur in both age groups [8,12,42]. Additionally, in a cadaveric laboratory study conducted by Thome and colleagues [42], a direct comparison was made between suture fixation and screw fixation in both skeletally mature and immature specimens. The findings indicated that in skeletally mature individuals, suture fixation may be a preferable option. Conversely, in skeletally immature individuals, suture fixation was found to be comparable to screw fixation.

Finally, though Type III and Type IV fractures usually necessitate surgery, in recent years, a growing body of evidence favors surgical intervention also for Type II fractures, especially in the presence of significant anterior lifting, to mitigate the risk of residual laxity and impingement [8,12,13]. In a survey conducted by Adams et al. [8], twenty orthopedic surgeons evaluated 40 cases of Type II tibial eminence fractures and were asked to classify them as operative or nonoperative. The results showed that 85% of the cases were classified as operative, with the main determining factor being the extent of anterior lifting. Furthermore, over 64% of surgeons opted for operative treatment when the fracture fragment was displaced by ≥3.5 mm.

This study introduces a modified arthroscopic technique using suture fixation in a triangular shape formation for Type II and Type III tibial eminence fractures. The technique is straightforward and can be replicated using basic arthroscopy equipment. The study’s results suggest that this technique is safe, effective, and allows for early rehabilitation compared to other techniques used for tibial eminence fractures. Additionally, the triangular shape of the suture provides better control of the bone fragment compared to double suture fixation and achieves anatomical reduction.

## 5. Limitations

We acknowledge some limitations in the study. The patient population is relatively small but should be understood given the rarity of high-grade tibial eminence fractures, which is consistent with existing literature. Furthermore, the post-hoc power analysis helps alleviate these concerns; the retrospective nature of a single-center study poses a limitation per se, though it was mitigated by the novelty of the suture fixation method and the selective inclusion of Type II tibial eminence fractures. In addition, the benefits of suture fixation have been described for quite some time but without the emphasis on the pull-through triangular fixation that includes an anchoring maneuver, as laid out in our current study. While future studies should include a control group to compare this technique with other fixation methods directly, the pull-through technique’s added value should still be acknowledged in the literature. Finally, postoperative anterior knee laxity was assessed subjectively by the surgeon and self-reported by the patient with objective measures. While an objective evaluation could potentially enhance the results, we firmly believe that the combination of patients’ subjective reports and questionnaires, along with clinical assessment, holds greater significance.

## 6. Conclusions

The study introduces a modified arthroscopic technique using triangular suture fixation for Type II and Type III tibial eminence fractures. This method has proven effective and safe, ensuring anatomical reduction, efficient and good control of the bone fragment, and fracture stability, with minimal surgical complications and no need for subsequent surgeries to remove hardware. The rehabilitation protocol enabled patients to regain functional range of motion and strength, facilitating an efficient return to pre-injury activity levels. This technique provides surgeons with an additional tool for managing these fractures, and it should be considered when treating these types of fractures.

## Figures and Tables

**Table 1:** Patient demographics.

		*n* (%)	Range
Age at surgery (SD), years		33.9 (8.4)	21–56
Gender	Male (%) Female (%)	8 (34.5) 15 (65.5)	
Laterality	Right (%) Left (%)	13 (56.25) 10 (44)	
Classification (%)	2	14 (60)	
	3	9 (40)	
Mechanism of injury (%)	Motorcycle accident	10 (43)	
	Bicycle accident	2(8)	
	Scooter accident Ski accident	1 (4) 8 (34)	
	Fall	2 (8)	
Time to surgery (days) (SD)		6.9 (4.1)	1–15
Follow-up, months		35.4	27–53
Union (SD)		23 (100)	
second arthroscopy- arhrolysis, *n*		5 (21)	
Time to second surgery, months (SD)		9.1 (5.5)	3.6–14.6
ROM flexion, degree (SD)		123 (16)	90–150
ROM extension, degree (SD)		−0.5 (−3.4)	−10 (0)
Collateral stability		0 *	
Lachman		0 *	
IKDC (SD)		72.7 (23)	23–100
Tegner Lysholm Knee Score (SD)		81.9 (17.4)	(68–100)

SD, standard deviation; ROM, range of motion; IKDC, International Knee Documentation Committee. * The value of zero indicates the absence of any abnormal or pathological findings.

## Data Availability

The data presented in this study are available on request from the corresponding author.

## Abbreviations

CT	Tomographic scans
MRI	Magnetic Resonance Images
ROM	Range Of Motion
ORIF	Open Reduction and Internal fixation
ARIF	Arthroscopic Reduction and Internal Fixation

## References

[1] Atsumi S., Arai Y., Nakagawa S., Inoue H., Ikoma K., Fujiwara H., Kubo T. (2016). A Case of Nonunion Avulsion Fracture of the Anterior Tibial Eminence. Case Rep. Orthop..

[2] Hargrove R., Parsons S., Payne R. (2004). Anterior tibial spine fracture—An easy fracture to miss. Accid. Emerg. Nurs..

[3] Kieser D.C., Gwynne-Jones D., Dreyer S. (2011). Displaced tibial intercondylar eminence fractures. J. Orthop. Surg..

[4] Lubowitz J.H., Elson W.S., Guttmann D. (2005). Part II: Arthroscopic treatment of tibial plateau fractures: Intercondylar eminence avulsion fractures. Arthroscopy.

[5] Najdi H., Thévenin-Lemoine C., Sales de Gauzy J., Accadbled F. (2016). Arthroscopic treatment of intercondylar eminence fractures with intraepiphyseal screws in children and adolescents. Orthop. Traumatol. Surg. Res..

[6] Meyers M.H., McKeever F.M. (1959). Fracture of the intercondylar eminence of the tibia. J. Bone Jt. Surg. Am..

[7] Zaricznyj B. (1977). Avulsion fracture of the tibial eminence: Treatment by open reduction and pinning. J. Bone Joint Surg. Am..

[8] Adams A.J., O’Hara N.N., Abzug J.M., Aoyama J.T., Ganley T.J., Carey J.L., Cruz A.I., Ellis H.B., Fabricant P.D., Tibial Spine Research Group (2019). Pediatric Type II Tibial Spine Fractures: Addressing the Treatment Controversy With a Mixed-Effects Model. Orthop. J. Sports Med..

[9] Baxter M.P., Wiley J.J. (1988). Fractures of the tibial spine in children. An evaluation of knee stability. J. Bone Joint Surg. Br..

[10] Gans I., Baldwin K.D., Ganley T.J. (2014). Treatment and Management Outcomes of Tibial Eminence Fractures in Pediatric Patients: A Systematic Review. Am. J. Sports Med..

[11] Jackson T.J., Storey E.P., Ganley T.J., Tibial Spine Interest Group (2019). The Surgical Management of Tibial Spine Fractures in Children: A Survey of the Pediatric Orthopaedic Society of North America (POSNA). J. Pediatr. Orthop..

[12] Prasad N., Aoyama J.T., Ganley T.J., Ellis H.B., Mistovich R.J., Yen Y.-M., Fabricant P.D., Green D.W., Cruz A.I. (2021). A Comparison of Nonoperative and Operative Treatment of Type 2 Tibial Spine Fractures. Orthop. J. Sports Med..

[13] Scrimshire A.B., Gawad M., Davies R., George H. (2018). Management and outcomes of isolated paediatric tibial spine fractures. Injury.

[14] Bonin N., Jeunet L., Obert L., Dejour D. (2007). Adult tibial eminence fracture fixation: Arthroscopic procedure using K-wire folded fixation. Knee Surg. Sports Traumatol. Arthrosc..

[15] McLennan J.G. (1982). The role of arthroscopic surgery in the treatment of fractures of the intercondylar eminence of the tibia. J. Bone Jt. Surg. Br..

[16] Davies E.M., McLaren M.I. (2001). Type III tibial spine avulsions treated with arthroscopic Acutrak screw reattachment. Clin. Orthop. Relat. Res..

[17] Doral M.N., Atay O.A., Leblebicioğlu G., Tetik O. (2001). Arthroscopic fixation of the fractures of the intercondylar eminence via transquadricipital tendinous portal. Knee Surg. Sports Traumatol. Arthrosc..

[18] Delcogliano A., Chiossi S., Caporaso A., Menghi A., Rinonapoli G. (2003). Tibial intercondylar eminence fractures in adults: Arthroscopic treatment. Knee Surg. Sports Traumatol. Arthrosc..

[19] Kobayashi S., Terayama K. (1994). Arthroscopic reduction and fixation of a completely displaced fracture of the intercondylar eminence of the tibia. Arthroscopy.

[20] Chang C.J., Huang T.C., Hoshino Y., Wang C.-H., Kuan F.-C., Su W.-R., Hong C.-K. (2022). Functional Outcomes and Subsequent Surgical Procedures After Arthroscopic Suture Versus Screw Fixation for ACL Tibial Avulsion Fractures: A Systematic Review and Meta-analysis. Orthop. J. Sports Med..

[21] Huang T.W., Hsu K.Y., Cheng C.Y., Chen L.-H., Wang C.-J., Chan Y.-S., Chen W.-J. (2008). Arthroscopic suture fixation of tibial eminence avulsion fractures. Arthroscopy.

[22] Jaramillo Quiceno G.A., Arias Pérez R.D., Herrera Mejía A.M. (2021). Satisfactory clinical outcomes using a novel arthroscopic technique for fixation of tibial spine avulsion fractures: Technical note. J. ISAKOS.

[23] Kelly S., DeFroda S., Nuelle C.W. (2023). Arthroscopic Assisted Anterior Cruciate Ligament Tibial Spine Avulsion Reduction and Cortical Button Fixation. Arthrosc. Technol..

[24] Lehman R.A., Murphy K.P., Machen M.S., Kuklo T.R. (2003). Modified arthroscopic suture fixation of a displaced tibial eminence fracture. Arthroscopy.

[25] Yuan L., Shi R., Chen Z., Ding W., Tan H. (2022). The most economical arthroscopic suture fixation for tibial intercondylar eminence avulsion fracture without any implant. J. Orthop. Surg. Res..

[26] Faivre B., Benea H., Klouche S., Lespagnol F., Bauer T., Hardy P. (2014). An original arthroscopic fixation of adult’s tibial eminence fractures using the Tightrope^®^ device: A report of 8 cases and review of literature. Knee.

[27] Lysholm J., Tegner Y. (2007). Knee injury rating scales. Acta Orthop..

[28] Abulhasan J.F., Snow M.D., Anley C.M., Bakhsh M.M., Grey M.J. (2016). An Extensive Evaluation of Different Knee Stability Assessment Measures: A Systematic Review. J. Funct. Morphol. Kinesiol..

[29] Kopkow C., Lange T., Hoyer A., Lützner J., Schmitt J. (2018). Physical tests for diagnosing anterior cruciate ligament rupture. Cochrane Database Syst. Rev..

[30] Tegner Y., Lysholm J. (1985). Rating systems in the evaluation of knee ligament injuries. Clin. Orthop. Relat. Res..

[31] Irrgang J.J., Anderson A.F., Boland A.L., Harner C.D., Kurosaka M., Neyret P., Richmond J.C., Shelborne K.D. (2001). Development and validation of the international knee documentation committee subjective knee form. Am. J. Sports Med..

[32] Schmitt L.C., Paterno M.V., Huang S. (2010). Validity and internal consistency of the international knee documentation committee subjective knee evaluation form in children and adolescents. Am. J. Sports Med..

[33] Hunter R.E., Willis J.A. (2004). Arthroscopic fixation of avulsion fractures of the tibial eminence: Technique and outcome. Arthroscopy.

[34] Chu Y., Hu T., Chen M., Jiang C., Wu Z., Shi J. (2021). Preliminary clinical outcomes of the double-row anchor suture-bridge technique for the fixation of tibial intercondylar eminence fractures in adults: A 12-months minimal follow-up. BMC Musculoskelet. Disord..

[35] Osti L., Buda M., Soldati F., Del Buono A., Osti R., Maffulli N. (2016). Arthroscopic treatment of tibial eminence fracture: A systematic review of different fixation methods. Br. Med. Bull..

[36] Yıldırım A., Aydın B.K., Çiftci S., Güleç A. (2020). Arthroscopic treatment of tibial eminence fractures using double-loop endobutton device: Surgical technique and short-term treatment outcomes. Jt. Dis. Relat. Surg..

[37] Koukoulias N.E., Germanou E., Lola D., Papavasiliou A.V., Papastergiou S.G. (2012). Clinical outcome of arthroscopic suture fixation for tibial eminence fractures in adults. Arthroscopy.

[38] Willis R.B., Blokker C., Stoll T.M., Paterson D.C., Galpin R.D. (1993). Long-term follow-up of anterior tibial eminence fractures. J. Pediatr. Orthop..

[39] Montgomery K.D., Cavanaugh J., Cohen S., Wickiewicz T.L., Warren R.F., Blevens F. (2002). Motion complications after arthroscopic repair of anterior cruciate ligament avulsion fractures in the adult. Arthroscopy.

[40] Zhang Q., Yang J., Zhao G., Zheng D., Zhou X., Xu N., Wang Y. (2017). A new technique for arthroscopic reduction and fixation of displaced tibial intercondylar eminence fractures, using suture anchor and EndoButton system. J. Orthop. Surg..

[41] Limone B., Zambianchi F., Cacciola G., Seracchioli S., Catani F., Tarallo L. (2023). Management and Outcomes of Tibial Eminence Fractures in the Pediatric Population: A Systematic Review. Children.

[42] Thome A.P., O’Donnell R., DeFroda S.F., Cohen B.H., Cruz A.I., Fleming B.C., Owens B.D. (2021). Effect of Skeletal Maturity on Fixation Techniques for Tibial Eminence Fractures. Orthop. J. Sports Med..

